# Knowledge, Attitude, and Barriers Influencing Seasonal Influenza Vaccination Uptake

**DOI:** 10.1155/2020/7653745

**Published:** 2020-10-16

**Authors:** Mazin A. Barry, Khalid I. Aljammaz, Abdulaziz A. Alrashed

**Affiliations:** ^1^Division of Infectious Diseases, Faculty of Medicine, King Saud University, Riyadh, Saudi Arabia; ^2^College of Medicine, Shaqra University, Shaqra, Saudi Arabia

## Abstract

**Background:**

Seasonal influenza is an acute respiratory infection caused by influenza viruses that are highly contagious and circulate in all parts of the world. It gives rise to an estimated 3 to 5 million cases of severe illness and about 250,000 to 500,000 deaths globally each year. Influenza tends to cause epidemics with serious illness and death among high-risk groups such as children aged 5 years and younger, pregnant women, elderly ≥65 years of age, and with chronic medical conditions. According to the Centers for Disease Prevention and Control (CDC), all people who are 6 months old and above are recommended to receive the seasonal influenza vaccine annually. Despite the fact that influenza vaccine is readily available, and the severity of the disease is known to adversely affect the individual's quality of life and well-being, vaccination uptake rates are still low, contributing to the increased burden of the disease worldwide.

**Objectives:**

To measure the influenza vaccine uptake among residents of Riyadh Province, Saudi Arabia, that determines their attitude, knowledge, and beliefs regarding the vaccine.

**Methods:**

A cross-sectional study was conducted using a self-administered structured questionnaire distributed online targeting residents of Riyadh Province, Saudi Arabia, from 1st of August 2019 till 30th of September 2019. Participants were selected through volunteer sampling. The questionnaire included demographic data including age, gender, occupation, education level, marital status, and comorbidities. It also included questions regarding knowledge, attitude, and beliefs regarding influenza vaccine. After collection of data, statistical analyses were conducted by using Statistical Package for Social Sciences (SPSS) version 19.0. A *P* value of <0.05 was considered statistically significant.

**Results:**

Our study included 503 participants, with age ranging from 18 to 65 years old and 324 (64%) were females. 100 participants (19.9%) had comorbid conditions, and 223 (44.3%) have been vaccinated against influenza in the past. A large portion of participants (41.2%) were familiar with seasonal influenza vaccination from the media. The knowledge part of the questionnaire showed that 302 (60%) participants knew how often they should receive the vaccine and 313 (62.2%) participants knew that the vaccine is provided freely in all of Saudi Arabia. In terms of belief and attitude, 371 participants (73.8%) thought they were susceptible to the disease and 365 (73.8%) believed that influenza vaccine is beneficial, while 446 participants (88.7%) thought that the general public need more knowledge and awareness on the scientific facts of influenza vaccine. Regarding barriers, 295 participants (58.6%) wanted to avoid vaccines and 252 (50.1%) were concerned about the vaccine's adverse effects. Participants with frequent health checkups and those who had previous knowledge on the availability of the vaccine for free were more likely to be vaccinated. Vaccinated participants (44.3%) were asked if they were willing to take the vaccine again when it is due, 158 (70.9%) answered yes. Those who elicited symptomatic reaction to the vaccine (26.0%) were less inclined to take it again (*P* = 0.035).

**Conclusion:**

We concluded that there is a low influenza vaccine uptake rate among our study population, considering that the barriers most commonly chosen by participants are solvable with health education and campaigns oriented towards delivering facts about the vaccine and dispelling misinformation; such measures are highly recommended and are postulated to carry a great benefit that should target common misconceptions identified in this study.

## 1. Introduction

Seasonal influenza is an acute respiratory infection caused by influenza viruses which are highly contagious and circulate in all parts of the world. It gives rise to an estimated 3 to 5 million cases of severe illness and about 250,000 to 500,000 deaths globally each year [1]. Despite the fact that influenza vaccine is readily available and the severity of the disease is known to adversely affect the individual's quality of life and well-being, vaccination uptake rates are still low, contributing to the increasing burden of the disease worldwide [2].

Influenza tends to cause epidemics with serious illness and death among high-risk groups such as children aged 5 years and younger, pregnant women, elderly ≥65 years of age, individuals with chronic medical conditions, and can cause infections among health care personnel [3]. According to the Centers for Disease Prevention and Control (CDC), all people who are 6 months old and above are recommended to receive the seasonal influenza vaccine annually [4]. The primary type of vaccine is an inactivated vaccine containing a killed virus strain, which is administered intramuscularly.

According to the World Health Organization (WHO), influenza vaccination coverage was <1% in most parts of Africa and Asia [5]. There are various studies in Middle East countries with vaccination uptake rates quite changeable according to country and time of year [5]. In Jordan, a study among the Jordanian population reported a low vaccination rate [6]. Furthermore, another study in Jordan targeting the community as a whole reported the overall vaccination rate ranging between 9.9% and 27.5% [7]. In addition, a study in Lebanon on the general adult population reported that the overall vaccination rate was 27.6% [8]. However, in Saudi Arabia, a study in the central region among military personnel reported a vaccine coverage rate of only 17.8% [9]. In addition, a study in Makkah conducted on adults visiting a primary health care center reported a vaccination rate of only 18.5% [10]. Various studies reported multiple factors influencing vaccination uptake including knowledge and attitude contributed to the individual's opinions [11]. In a study in Lebanon, “thinking that the vaccine was not needed” was the only factor associated with lack of receiving the annual influenza vaccine [8]. Concerns about its safety and efficacy were the most reported barriers [6]. In addition, a German study conducted on the general public reported that “fear of side effects” and “vaccination was not necessary” were the most reported concerns influencing influenza vaccine uptake [12].

The Assistant Agency for Preventive Medicine of the Saudi Ministry of Health is responsible for influenza surveillance in the country [13], and as Saudi Arabia has its unique situation with the annual pilgrimage, when millions of people travel to the holy cities of Mecca and Madinah, the Saudi Thoracic Society has published guidelines for influenza vaccination [14].

In our study, we hypothesized that the general knowledge on influenza vaccination and its benefits in our target population was lacking, and the attitude towards it is inadequate, manifesting in unwillingness to uptake or indifference towards it due to unawareness of possible influenza-related morbidity/mortality that might arise from vaccine ignorance or avoidance.

We also postulated that different barriers may exist such as fear of side effects that are rumored in social media without scientific evidence. Our target was the population of Riyadh Province in the Kingdom of Saudi Arabia. We aimed to determine the knowledge, attitude, and barriers influencing seasonal influenza vaccine uptake.

## 2. Methods

### 2.1. Research Design and Setting

A cross-sectional study was conducted in Riyadh Province, Saudi Arabia, from 1st of August 2019 till 30th of September 2019. The study was carried out by distributing an online self-administered semistructured questionnaire through SurveyMonkey. The study was approved by Institutional Review Board (IRB) of King Saud University-College of Medicine (no. E-19-4115).

### 2.2. Sample Size

We calculated the sample size relying on this established formula *n* = (*z*^2^ × *p* × *q*)/*d*^2^, where *n* = the minimum sample size, *z* = the confidence level (1.96), *p* = variability of the population (0.5), *q* = (1 − *p*) = (0.5), and *d* = margin of error = (0.05). After applying the formula *n* = (*z*^2^ × *p* × *q*)/*d*^2^. We found that the minimum sample size to achieve a precision of ±5% with a 95% confidence interval (CI) is 384.

### 2.3. Development and Application of Questionnaire

Surveys that were used previously and published [6, 15] were used to design our questionnaire that included three parts. The first part included demographic characters including age, gender, occupation, education level, marital status, and comorbidities with emphasis on participant source of information regarding seasonal influenza and its vaccine. The second part included questions regarding participant's knowledge and attitude. Participants were asked about the vaccine availability, side effects, route of administration, and how often the vaccine should be taken. In addition, it also included the following question: is the vaccine advisable for all people? The third part included questions regarding barriers towards influenza vaccine such as its safety and effectiveness.

### 2.4. Procedure

The questionnaire was distributed online in social media and external websites. Participants were asked to give their consent before starting their answers.

### 2.5. Statistical Analysis

IBM SPSS version 19 was utilized for data entry, management, and analysis. Descriptive statistics were utilized in the form of frequencies and percentages for categorical data, mean of standard deviation for quantitative data if it is normally distributed, or median of quartiles if data are skewed. To compare qualitative variables, we utilized the chi-square test, and we used *P* < 0.05 to determine statistical significance.

## 3. Results

Out of 712 respondents, 503 participants completed the full survey (70% response rate), age ranged from 18 to 65 years old, 324 (64%) were females, and 273 (54%) were single. University level of education or above was recorded among 356 (70%) of the sampled population and 169 (33.6%) were students. 100 participants (19.9%) had comorbid conditions such as hypertension, diabetes mellitus, asthma, chronic obstructive lung disease, and congestive heart failure, 223 (44.3%) have been vaccinated against influenza in the past. 207 (41.2%) knew about seasonal influenza vaccination through a family member, 117 (23.3%) from television, 90 (17.9%) from the Internet, and only 76 (15.1%) from health care providers.

The knowledge part of the questionnaire showed that 302 (60%) of participants knew how often they should receive the vaccine, 209 (41.6%) knew about the primary vaccine route of administration, 313 (62.2%) knew that the vaccine is provided freely in all of Saudi Arabia, and only 84 (16.7%) knew that elderly population with or without comorbidities are indicated for seasonal influenza vaccination (Table 1).

Regarding the belief and attitude of participants, 371 (73.8%) thought they were susceptible to the disease and 365 (73.8%) believed that influenza vaccine is beneficial. However, 250 (49.7%) believed that seasonal influenza is not serious enough to warrant vaccination. 446 (88.7%) think that the general public need more knowledge to be aware of the scientific facts on influenza vaccine.

When asked about their opinion on the barriers to seasonal influenza vaccination uptake, the main barrier among 295 participants (58.6%) was that they want to avoid vaccinations, 252 (50.1%) were concerned about the vaccine's adverse effects, 205 (40.8%) believed that the vaccine had no benefit, 195 (38.8%) thought that the vaccine was unsafe, 229 (45.5%) thought they were not considered within a high-risk group and therefore do not need vaccination, 232 (46.1%) did not know that the vaccine was useful, and lastly 190 (37.8%) do not have time to get vaccinated (Table 2).

Using the bivariate analysis method to demonstrate the relation of participant's knowledge, attitude, and overall characteristics to vaccination uptake, it was shown that participants with frequent health checkups (at least once every 6 months) are more likely to be vaccinated (*P*=0.001) than those who do not have frequent follow-ups. Participants who were knowledgeable about the free-of-charge vaccine availability had more vaccination uptake rates (*P*=0.001). Vaccinated participants (223) were more likely to choose the correct answer when asked about the vaccine route of administration and frequency (*P*=0.001). There was no statistically significant relation between vaccine uptake and age, gender, marital status, education level, or presence of comorbidities (Table 3).

Regarding general knowledge of seasonal influenza, 320 (63.6%) knew of its symptoms and were able to list them. 420 (83.5%) knew its mode of transmission, and 348 (69.2%) identified it as a respiratory disease.

The most common chosen reasons for vaccine uptake were as follows: 92 (41.3%) got vaccinated because they believed that the vaccine was beneficial, 62 (27.8%) took it as part of the ministry of health's advice, 35 (15.7%) took it to protect their close loved ones and others, while only 34 (15.2%) participants took it to perform the Hajj pilgrimage.

The reported vaccination-related adverse events in a descending order were fever 24 (41.4%), redness at the injection site 21 (36.2%), throat congestion 11 (19%), and generalized aches 2 (3.4%).

Finally, vaccinated participants 223 (44.3%) were asked if they were willing to take the vaccine again when it is due, 158 (70.9%) answered yes. Those 58 (26%) who elicited symptomatic reaction to the vaccine were less inclined to take it again (*P*=0.035).

## 4. Discussion

Our study included 503 participants with age ranging from 18 to 65 years old. Females had more presence than males, representing 64% of participants, which is a common finding in cross-sectional online surveys in the region. Participants who reported comorbid conditions (20%) reported being previously vaccinated less than half of the time (44.3%), which is far from what was found in another study conducted on participants with type 2 diabetes mellitus in the region where 61% of participants were vaccinated [16].

In this study, while associating participant's characteristics with influenza vaccine uptake, it was found that 56.6% of participants aged 36 or older do not receive annual influenza vaccine. Participants with regular health checkups (55.3%) were more likely to get vaccinated than their counterparts, most likely due to the influence of the health care providers even though only 15.1% reported so. Knowledge about free vaccine availability had a statistically significant relation to vaccine uptake, and 124 (65.3%) of participants who did not know this fact were unvaccinated.

As expected, participants who were previously vaccinated are more likely to correctly answer the route of administration (125 or 59.8%) and frequency of vaccination (160 or 53.0%).

The most common concern among the contributors in this study regarding the barriers to influenza vaccine was that they want to avoid vaccinations (58.6%), which is relatively close to a previous study conducted in Riyadh city in which 149 out of 207 (72%) participants gave the same reason [15].

A large portion of participants (61.2%) thought that the vaccine was unsafe, and 59.2% thought it did not provide benefit, which is similar to another study conducted in 2015 in Saudi Arabia [10].

When assessing the risk group's relation with vaccine uptake, it was found that 45.5% of participants think that they are not within the high-risk population in need of vaccination, a result different from that reported five years ago which showed that 90.8% of the contributors thought that they were not part of a high-risk group recommended for vaccination [10]. This discrepancy can be attributed to the new wave of awareness campaigns and the ministry of health's effort to educate the public about influenza vaccine.

One of the factors that influenced the refusal of getting vaccinated in a study done in Turkey was concern about side effects among 67.2% of health-care workers; this study, though in community, showed that only 50% avoid the vaccine due to this reason [17]. On the other hand, a study done in China among health-care workers showed that only 14.3% worried about theses adverse effects [18].

A study published in 2018 conducted on patients visiting primary health-care clinics showed that one-third of patients thought influenza is a simple disease and there was no need to prevent it or vaccinate against it, half of them thought their chances of getting the disease are low, and a third do not believe the vaccine is effective. Almost half of our study population believes in all three [15]. In Germany specifically, a study was conducted to evaluate the reason for low vaccine uptake in patients with comorbid illnesses. When patients were asked about their knowledge on influenza vaccine necessity for people with comorbidities, 21.2% felt that their chances of getting the disease were low [19].

In regards to time, 37.8% of participants expressed lack of free time to get vaccinated which was a slightly close result when compared to a study done in Southern California showing 44.9% of participants agreeing that they do not have time to get a flu shot [20].

In the knowledge and attitude section, we found that 62.2% knew about the availability of vaccine in health centers, 73.8% believed they were susceptible to seasonal influenza, 72.6% believed it provides benefit, while the majority (88.7%) think that the general population is in need of more campaigns and awareness about the vaccine. Only 16.7% answered correctly that the vaccine is not contraindicated for elderly people with comorbidities. These percentages were all comparable to other previous studies [15].

Finally, the main reasons for vaccination avoidance found by a previous Saudi study were misconception that the vaccine causes influenza (38.5%) and concern about vaccine efficacy (32.7%), while in this study the main reasons were avoiding vaccinations and fear of side effects [21]. In light of this, we can postulate that increasing vaccine uptake rates can be achieved by raising the community's knowledge and awareness regarding seasonal influenza and its vaccination by more extensive flu campaigns and education.

## 5. Conclusion

We have concluded that there is a low influenza vaccine uptake rate among the study population, considering that the barriers most commonly chosen by participants are solvable with health education and campaigns oriented towards delivering facts about vaccine and dispelling misinformation; these are highly recommended and postulated to carry a great benefit and should target common misconceptions identified in this study, with innovative solutions to gain quick access for vaccination to address issues with lack of time for some individuals.

## Figures and Tables

**Table 1:** Knowledge and attitude, *n* (%).

How often do you think influenza vaccines should be taken? (*n* = 503)
Correct, 302 (60%)
Wrong, 201 (40%)

Did you know about the availability of the vaccine in health centers this season? (*n* = 503)
Yes, 313 (62.2%)
No, 190 (37.8%)

How is the influenza vaccine administered? (*n* = 503)
Correct, 209 (41.6%)
Wrong, 294 (58.4%)

Is the influenza vaccine contraindicated for people of old age and comorbidities? (*n* = 503)
Correct, 84 (16.7%)
Wrong, 419 (83.3%)

Do you believe you are susceptible to seasonal flu? (*n* = 503)
Yes, 371 (73.8%)
No, 132 (26.2%)

Do you believe the influenza vaccine provides benefits? (*n* = 503)
Yes, 365 (72.6%)
No, 138 (27.4%)

Do you think the general public is in need of more awareness about the vaccine? (*n* = 503)
Yes, 446 (88.7%)
No, 57 (11.3%)

**Table 2:** Barriers to seasonal influenza vaccine, *n* (%).

I want to avoid medications
Agree to strongly agree, 295 (58.6%)
Disagree to strongly disagree, 208 (41.4%)

I believe seasonal flu is not serious enough to warrant vaccination
Agree to strongly agree, 250 (49.7%)
Disagree to strongly disagree, 253 (50.3%)

I am concerned about the vaccine side effects
Agree to strongly agree, 252 (50.1%)
Disagree to strongly disagree, 251 (49.9%)

I think the influenza vaccine does not provide benefit
Agree to strongly agree, 205 (40.8%)
Disagree to strongly disagree, 298 (59.2%)

I think the influenza vaccine is unsafe
Agree to strongly agree, 195 (38.8%)
Disagree to strongly disagree, 308 (61.2%)

I think the chance of getting seasonal flu is low
Agree to strongly agree, 232 (46.1%)
Disagree to strongly disagree, 271 (53.9%)

I do not think I'm one of the primary high-risk populations that needs vaccination
Agree to strongly agree, 229 (45.5%)
Disagree to strongly disagree, 274 (54.5%)

I did not know that influenza vaccine is useful
Agree to strongly agree, 232 (46.1%)
Disagree to strongly disagree, 271 (53.9%)

I don't have the time to get vaccinated
Agree to strongly agree, 190 (37.8%)
Disagree to strongly disagree, 313 (62.2%)

**Table 3:** Participant's characteristics' association with influenza vaccine uptake.

Characteristic	Vaccinated (*n* = 223)	Unvaccinated (*n* = 280)	*P* value
*Age:*			0.748
>36	75 (43.4%)	98 (56.6%)	
<36	148 (44.8%)	182 (55.2%)	

Sex—M : F (%)	87 (48.6%) : 136 (42.0%)	92 (51.4%) : 188 (58.0%)	0.152

*Marital status* (married)	95 (41.3%)	135 (58.7%)	0.209
*Education level*			0.157
School	58 (39.5%)	89 (60.5%)	
University or above	165 (46.3%)	191 (53.7%)	
People with comorbidity	51 (51.0%)	49 (49.0%)	0.134

*Frequent health checkups*			0.001^†^
Yes	115 (55.3%)	93 (44.7%)	
No	108 (36.6%)	187 (63.4%)	

*Knowledgeable about free vaccine availability*			0.001^†^
Yes	157 (50.2%)	156 (49.8%)	
No	66 (34.7%)	124 (65.3%)	

*Knowledgeable about route of administration*			0.001^†^
Yes	125 (59.8%)	84 (40.2%)	
No	98 (33.3%)	196 (66.7%)	

*Knowledgeable about frequency*			0.001^†^
Yes	160 (53.0%)	142 (47.0%)	
No	63 (31.3%)	138 (68.7%)	

A *P* value of <0.05 is considered statistically significant. Percentages are the sum of their respective characteristic. ^†^Statistically significant.

## Data Availability

All data are included within the manuscript.

## References

[1] WHO (2014). Seasonal influenza fact sheet.

[2] WHO (2015). *The Global Action Plan for Influenza Vaccines Report of the Tenth Meeting of the Advisory Group of the WHO Global Action Plan for Influenza Vaccines*.

[3] World Health Organization (2016). Influenza (seasonal) fact sheet number 2112014. http://www.who.int/mediacentre/factsheets/fs211/en/.

[4] Grohskopf L. A., Sokolow L. Z., Broder K. R. (2017). Prevention and control of seasonal influenza with vaccines: recommendations of the advisory committee on immunization practices-United States, 2017-2018 influenza season. *American Journal of Transplantation*.

[5] World Health Organization (2015). *Global Influenza Programme Seasonal Influenza Vaccine Use in Low and Middle Income Countries in the Tropics and Subtropics–A Systematic Review*.

[6] Abu-Rish E. Y., Elayeh E. R., Mousa L. A., Butanji Y. K., Albsoul-Younes A. M. (2016). Knowledge, awareness and practices towards seasonal influenza and its vaccine: implications for future vaccination campaigns in Jordan. *Family Practice*.

[7] Assaf A. M., Hammad E. A., Haddadin R. N. (2016). Influenza vaccination coverage rates, knowledge, attitudes, and beliefs in Jordan: a comprehensive study. *Viral Immunology*.

[8] El Khoury G., Salameh P. (2015). Influenza vaccination: a cross-sectional survey of knowledge, attitude and practices among the Lebanese adult population. *International Journal of Environmental Research and Public Health*.

[9] Al-Khashan H. I., Selim M. A., Mishriky A. M., Binsaeed A. A. (2011). Meningitis and seasonal influenza vaccination coverage among military personnel in central Saudi Arabia. *Saudi Medical Journal*.

[10] Korani M. (2015). Assessment of seasonal flu immunization status among adult patients visiting al-Sharaee primary health care center in Makkah al-Mokarramah. *International Journal of Medical Science and Public Health*.

[11] Kravos A., Kračun L., Kravos K., Iljaž R. (2015). The impact of patient’s socio-demographic characterictics, comorbidities and attitudes on flu vaccination uptake in family practice settings/vpliv bolnikovih psihosocialnih značilnosti, komorbidnosti in stališč Na odločitev O cepljenju proti gripi V ambulantah družinske medicine. *Slovenian Journal of Public Health*.

[12] Bödeker B., Remschmidt C., Schmich P., Wichmann O. (2015). Why are older adults and individuals with underlying chronic diseases in Germany not vaccinated against flu? a population-based study. *BMC Public Health*.

[13] Assistant Agency for Preventive Medicine Ministry of Health, Influenza Surveillance in Saudi Arabia (2017). Assistant Agency for Preventive Medicine Ministry of Health. https://www.moh.gov.sa/CCC/healthp/regulations/Documents/ISSA%20Protocol.pdf.

[14] Stiver H. G. (2015). Influenza vaccination guidelines: a special case for Saudi Arabia. *Annals of Thoracic Medicine*.

[15] Sagor K., AlAteeq M. (2018). Beliefs, attitudes، and barriers associated with the uptake of the seasonal influenza vaccine among patients visiting primary healthcare clinics. *Saudi Medical Journal*.

[16] Alnaheelah I., Awadalla N., Al-Musa K., Alsabaani A., Mahfouz A. (2018). Influenza vaccination in type 2 diabetes patients: coverage status and its determinants in southwestern Saudi Arabia. *International Journal of Environmental Research and Public Health*.

[17] Savas E., Tanriverdi D. (2010). Knowledge, attitudes and anxiety towards influenza A/H1N1 vaccination of healthcare workers in Turkey. *BMC Infectious Diseases*.

[18] Ye L., Chen J., Fang T. (2018). Determinants of healthcare workers’ willingness to recommend the seasonal influenza vaccine to diabetic patients: a cross-sectional survey in Ningbo, China. *Human Vaccines & Immunotherapeutics*.

[19] Bödeker B., Remschmidt C., Schmich P., Wichmann O. (2015). Why are older adults and individuals with underlying chronic diseases in Germany not vaccinated against flu? a population-based study. *BMC Public Health*.

[20] Rogers C. J., Bahr K. O., Benjamin S. M. (2018). Attitudes and barriers associated with seasonal influenza vaccination uptake among public health students; a cross-sectional study. *BMC Public Health*.

[21] Haridi H. K., Salman K. A., Basaif E. A., Al-Skaibi D. K. (2017). Influenza vaccine uptake, determinants, motivators, and barriers of the vaccine receipt among healthcare workers in a tertiary care hospital in Saudi Arabia. *Journal of Hospital Infection*.

